# Effects of 11-Ketotestosterone on Development of the Previtellogenic Ovary in the Sterlet, *Acipenser ruthenus*

**DOI:** 10.3389/fendo.2020.00115

**Published:** 2020-03-25

**Authors:** Wei Wang, Hua Zhu, ZhaoHui Tian, Ai Sun, Ying Dong, Tian Dong, HongXia Hu

**Affiliations:** ^1^Beijing Fisheries Research Institute & Beijing Key Laboratory of Fishery Biotechnology, Beijing, China; ^2^National Freshwater Fisheries Engineering Technology Research Center, Beijing, China

**Keywords:** 11-Ketotestosterone, Vtg, previtellogenic ovary, sterlet, lipid synthesis

## Abstract

11-ketotestosterone (11-KT) is a non-aromatizable and the most potent androgen in a few teleost. It has been reported that 11-KT in serum had a high concentration and increased sharply before the period of yolk deposition in females of few fishes. The aim of this study was to analyze the role of 11-KT both *in vivo* and *in vitro* on ovarian development, related gene expression levels, Vitellogenin (Vtg) synthesis, and serum sex steroid concentrations in previtellogenic cultured sterlet (*Acipenser ruthenus*). Silastic strips embedded with 11-KT (5 or 25 mg/kg) were implanted *in vivo* for 30 days. Ovarian masculinization or sex reversal was not observed. Histological analysis showed that 11-KT promoted sterlet ovarian development in a dose-dependent manner. Vtg and testosterone (T) increased significantly, while 17β-estradiol (E2) decreased with no significant difference among groups. The expression of genes androgen receptor (*ar*), *vtg* and lipoprotein lipase (*lpl*) were significantly increased in liver. However, 11-KT had no effect on the expression of *foxl2* and *cyp19a1* in ovary. *In vitro*, after incubation with 11-KT (10 and 100 μM) for 5 days, both T and E2 concentrations increased in both hepatic explants and ovarian explants culture medium; the concentration of Vtg also increased in hepatic explants culture medium. The expression of *ar, era, vtg*, and *lpl* increased significantly in hepatic explants. However, only the expression of *era* significantly increased in cultured ovarian explants. Altogether, these results suggest that 11-KT induced ovarian development, as well as Vtg and lipid synthesis, and could be an important factor facilitating the initiation of Vtg synthesis in the liver of the previtellogenic sterlet.

## Introduction

Vitellogenin (Vtg) is a large lipoglycophosphoprotein that serves as the major precursor of egg yolk proteins in females from oviparous vertebrates, including fish (1). The synthesis of Vtg and the decomposition of lipids are the main activities in vitellogenesis stage (2). Vtg is synthesized in the liver, then released to the bloodstream and transported to the ovary, where it is cleaved into the lipoproteins and phosphoproteins to make up egg yolk, an essential nutrient for future embryogenesis (1, 2). In female teleost, vitellogenesis is mainly regulated by 17β-estradiol (E2), the main natural estrogen secreted by the ovarian follicle (3, 4). This steroid also dominates the early phase of the reproductive cycle, but is consistently low during testis development (5–9). Conversely, 11-ketotestosterone (11-KT) which is synthetized from testosterone (T) in Leydig cells (10), is the most potent and non-aromatizable androgen in a few male teleost (11). This potent steroid stimulates testis growth, spermatogenesis, and reproductive behavior (10).

Increasing studies suggest that androgens play an important role in oocytes maturation of mammal (12, 13) and fish (14, 15). 11-KT plays an important role on the growth of pre-vitellogenic occyte in few teleost (16, 17), such as Anguillidae, Acipenseridae, and Mugilidae. Immature female short-finned eels (*A. australis*) treated with 11-KT implanting (25 mg/kg) displayed maturation traits, such as “chisel-shaped” snouts, enlarged eyes' size, larger liver and gonad in which oocytes develop from stage II to III (18). 11-KT treatment increases pre-vitellogenic oocyte size and the number of oil droplets in the oocyte of Japanese eels (*Anguilla japonica*) (19–21) and New Zealand long-finned eels (*A. dieffenbachii*) (21). Primary (late perinucleolar stage) ovarian follicles of Coho Salmon (*Oncorhynchu kisutch*) increases significantly in size after treatment with 11-KT (0.003–30 ng/ml) *in vitro* (22). *In vivo* (0.05–5 mg/kg) and *in vitro* (0–1,000 μM), 11-KT and T induce previtellogenic oocyte growth and development, and these effects are more pronounced with 11-KT exposure in Atlantic cod (*Gadus morhua*) (23, 24).

Generally, plasma levels of 11-KT are considerably higher in males while T levels are similar between male and female teleost (25). However, in eel and sturgeon females, serum 11-KT concentrations is as high or higher than those of T (17). 11-KT is appeared to be synthetized within the ovary and concentrations are correlated to ovarian development in Siberian sturgeon (*Acipenser baeri*) (26). In the females of wild beluga (*Huso huso*), Russian sturgeon (*A. gueldenstaedtii*), stellate sturgeon (*A. stellatus*), and European sturgeon (*A. sturio*), serum 11-KT concentrations were high during oocyte maturation, especially during vitellogenesis (27–29). Moreover, in the 7-year old Siberian sturgeon there is a gradual increase of 11-KT paired with E2 serum concentrations during the ovarian development (30). In previtellogenic Amur sturgeon (*A. schrenckii*) females, 11-KT concentrations were no less than that in the male fish at the beginning of the yolk deposition (31). Those results suggest that 11-KT can be the main active androgen in female sturgeon and play an important role in ovarian development, especially before yolk deposition. However, it remains unclear whether 11-KT is the critical factor inducing the beginning of the yolk deposition phase, and this requires further investigation.

There are some differences in the time of sexual maturity and gonadal development cycle among different sturgeon species (30). The gonadal development of female fish is not synchronous in natural and cultured environments. The development of female cultured sturgeon in stage II-III is often delayed, which is vitellogenesis and lasted for 1 year or even several years (5, 30, 31). Therefore, it is important to understand the role of 11-KT in ovarian development. Sterlets (*A. ruthenus*), the smallest and earliest sexual maturity species within the sturgeon family (32), were chosen for this study. In order to understand the effect of 11-KT on previtellogenic ovarian development and induction of the yolk deposition for previtellogenic sterlet, we conducted experiments *in vivo* and *in vitro*. *In vivo*, slow-release silastic strips embedded with 11-KT or unembedded were implanted for 30 days. *In vitro*, hepatic and ovarian explants from sterlet in ovary stage II were incubated for 5 days. Morphometrics, sex steroid hormones, Vtg and related gene expression were measured. These studies are beneficial to better understanding of the role of 11-KT in ovarian development, which is important for synchronizing ovarian development and promoting sexual maturity in the sturgeon hatcheries and breeding industry.

## Materials and Methods

### Animals and Synthetic 11-KT Powder

All the sampling sterlets were artificially propagated progeny from the Sturgeon Seed Farm of the State (Beijing, China). All animal handling and manipulation procedures were based on the standards of the Chinese Council on Animal Care. Before sampling, all samples were anesthetized with 400 ppm of MS222.

For these experiments, 11-KT powder was synthetized in Academy of Military Medical Sciences, through reducing C17 ketone group of adrenosterone (284998, Sigma) to hydroxyl with the catalytic reaction of sodium borohydride (33). Using standard 11-KT (K8250, Sigma) as control, the identification and purity of synthetized 11-KT was 99.9% checked by HPLC (High Performance Liquid Chromatography), and then stored at 4°C.

### Experiment 1: *in vivo* Effects of 11-KT on Ovarian Development, Target Gene Expression, Sex Steroid Concentrations and Vtg Synthesis

#### Manufacture of Slow-Release 11-KT Silastic Strips

Slow-release 11-KT Silastic Strips were made in the lab of Prof. Lin Hao-ran, Sun Yat-sen University. The dry 11-KT was mixed and thoroughly homogenized with unpolymerized medical elastomer base and coagulator silastic MDX4-4210. After uniform mixing, the paste was dried and processed into silastic strips (1.5 mm in diameter and 30 mm in length). Each strip carried 25 mg 11-KT. All strips were kept at 4°C in aluminum foil until use.

#### Animals and 11-KT Implantation

Twenty-eight-month old sterlets were randomly collected on Aug. 2015. After endoscopic detection under anesthesia, eighteen previtellogenic females were selected for implantation and divided into three balanced groups: one control group (355.30 ± 27.93 g, *n* = 6), two treatment groups consisting of a lower dose group (5 mg/kg, 375.12 ± 50.37 g, *n* = 6), and a high dose group (25 mg/kg, 405.83 ± 49.84 g, *n* = 6). No significant difference existed between groups (*P* = 0.142). Fish were fed with commercial standard diets daily (Zhongshan Presidents Co. LTD.).

After being anesthetized with 400 ppm of MS222, a small ventral midline incision was performed on all sterlets. In the treatment groups, the appropriate length of 11-KT silastic strips were cut and implanted to achieve the corresponding dose (5 or 25 mg/kg, respectively). In the control group, silastic strips devoid of 11-KT were implanted in an identical manner as in the treated groups. Following surgery, the incisions were daubed erythromycin ointment to prevent wound infection. Then, sterlets were transferred to indoor cylinder tanks (1 m^3^) and reared in flowing water for 30 days. Water temperature in the tanks ranged from 16.8 to 21.4°C.

#### Sampling

At the time of implantation start, as well as 15 and 30 days, about 1.5 mL of blood was drawn from the caudal vasculature of each anesthetic sterlet (*n* = 18 in every time point). Serum was separated by centrifugation at 4,000 g and 4°C for 10 min and stored in −80°C until analysis of the T, E2, and Vtg concentration.

Thirty days after implantation, fish were anesthetized with 400 ppm of MS222 (*n* = 18) and body weights, liver weights, and gonad weights of each sample were measured. The hepatosomatic index (HSI) was calculated by [(liver weight/ body weight) × 100]. Gonadosomatic index (GSI) was calculated by [(gonad weight/ body weight) × 100]. A portion from the central part of ovaries was fixed in Bouin's solution for histological observation. The liver and remaining ovary were temporarily preserved in RNAlater solution (AM7021, Ambion) and then kept at −80°C after being flash frozen by a plunge in liquid nitrogen.

### Experiment 2: *in vitro* Effects of 11-KT on Target Gene Expression, Sex Steroid Concentrations, and Vtg Synthesis in Hepatic and Ovarian Explants

A stock solution of 1,000 μM (3,000 ng/mL) 11-KT (MW = 302.408) was prepared by dissolving in 40 μL ethanol (0.16% of final incubation volume), and then added to 5 mL with DMEM/F12 (1:1, 1X, no phenol red Gibco 11039-21).

Three 28-month old female sterlets were selected through endoscopic determination at the previtellogenic stage. After being anesthetized with 400 ppm of MS222, the sterlets were briefly submerged in 75% ethanol prior to the removal of ovaries and liver under sterile conditions. A portion consistently resected from the central part of ovaries was fixed in Bouin's solution for histological analysis. Following adipose tissue removal, the remaining ovarian tissue and livers were washed separately by cold PBS (1X, PH = 7.4, HyClone, AAF203865) and cut into 1 cm^3^ fragments in culture medium (DMEM/F12, 1:1, 1X, phenol-red free, Gibco 11039-21). Using 6-well Costar culture plates, fragments were incubated in 2.5 mL culture medium for 5 days at 25°C. Three replicate incubations were performed for each treatment and for each individual. The culture medium consisted of DMEM/F12 (1:1, 1X, phenol-red free, Gibco 11039-21) supplemented with 1% penicillin-streptomycin solution (100X, Hyclone Sv30010), 20% fetal bovine serum (Gibco 10099-141) and 0, 10, 100 μM 11-KT (0, 30, or 300 ng/mL). At the end of incubation, explants were flash frozen in liquid nitrogen and stored at −80°C until analysis. Culture medium from each dish was also collected to determine T,E2, and Vtg concentrations.

### Histological Procedure

Fixed ovaries were dehydrated, paraffin embedded, and sectioned at 7 μm, and then stained with hematoxylin and eosin (Leica pathological section system) following standard procedures. Slides were observed with an optical microscope and digitally photographed (Olympus BX51). Classification of the gonadal development stage was based on Amiri et al. (9).

### Measurement of Sex Steroid Hormone and Vtg

About 0.8 mL of plasma was used for sex steroid hormone and Vtg concentration determination. According to manufacturer's instructions, Vtg was measured using the Vitellogenin Elisa Kit (V01003402, Biosense laboratories, Bergen, Norway). T was measured by the Testosterone Radioimmunoassay Kit (Cat. No. B10PZA, Beijing North Biotechnology Institute, Beijing, China). The minimum detectable limit of the method was 0.02 ng/ML for T. all antibodies had <0.1% cross-reactivity with closely-related steroids, such as dihydrotestosterone, 1.1 × 10^−2^%; 17β-estradiol, 2.1 × 10^−2^%; estriol, 6.2 × 10^−15^%; progesterone, 3.2 × 10^−2^%; and 11-KT, 1.2 × 10^−2^%. E2 was measured by chemiluminescent immunoassay using the Quantitative Determination Kit 17β-estradiol (Cat. No. YZB0051-2006, Beijing North Biotechnology Institute, Beijing, China). The minimum detectable limit of the method was <4.0 pg/ml for E2. Cross-reactivity of the antibody, as provided by the manufacturer, was as follows: estriol, <0.5%; progesterone <1.5%; and testosterone <0.01%. The standard curve was built with linear model logit (%B/B0) vs. log concentrations. All samples were analyzed in duplicate. Intra-assay variance averaged 8.38, 5.9, and 6.4% for Vtg, T, and E2, respectively, which fell within acceptable levels.

### RNA Isolation and cDNA Synthesis

Total RNA from tissues and explants was extracted by using RNeasyplus Mini Kit (Cat No. 74134, Qiagen). RNase-Free DNase Set (Cat No. 79254, Qiagen,) was used to remove genomic DNA during RNA extraction. RNA quantity, quality, and purity were measured by Nanodrop 2000 (Thermo, USA). Ribosomal 28s and 18S were used to assess RNA quality. All samples were deemed of high quality and were used for cDNA synthesis. Total RNA (4 μg) from each sample was simultaneously reverse-transcribed with an oligo(dT) primer using the SuperScript III First-Strand Synthesis System (Cat No.18080051, Invitrogen).

### Gene Cloning and Quantitative Real-Time PCR

Primers for *cyp19a1, foxl2, ar, era, erb* were obtained from Wang et al. (34). Other primers were designed using Primer 3 (http://bioinfo.ut.ee/primer3-0.4.0/primer3/). These primer sequences are detailed in Table 1. Specificity of the primer sets was confirmed by cDNAs cloned into pMD19-T vector (Takara) and sequencing by ABI 3730 (Tsingke Biological Technology). Primer efficiencies and gene expression were calculated according to Pfaffl (36).

**Table 1:** Primer sets used for quantitative real-time PCR.

**Gene**	**Primers sequence (5^**′**^-3^**′**^)**	**Amplicon size**	***R*^**2**^**	**Eff%**	**Accession no**
*foxl2*	F:AGTTTGCTTGCTCCCGTCA R:CGGTTGTCGTCGCTGTTT	154	0.991	97.62	KF870575
*cyp19a1*	F:GATAGCAGCACCTGACACCA R:TTTCCTCCAGCAGTTTCCTC	95	0.999	99.23	KP996196
*ar*	F:AAGCCAATCCTGTTTCACAA R:CACTGCCTGTTGACTAAATG	121	0.999	99.56	KP996194
*era*	F:GTGCCTTTCCCTTTCTCTCC R:GGCTTGTCTTCTGGACTGCT	146	0.999	96.40	KP996192
*erb*	F:ATTGCTGCTGGAGATGCTG R:TTCTGGCTTTGAACAGGTGA	121	0.998	99.41	KP996193
*vtg*	F:CTCAGGAACATCGCCAAGA R:TGCTGGGTCTGGTTTCAAG	130	0.996	101.65	KF918450
*vtgr*	F:TCTTGCTCATCCCTTTGCTC R:CTGTGGTTCATTCAGGTTGTTAG	138	0.997	96.57	KX247393
*lpl*	F:GCTCCATCCACCTGTTCATT R:CATCACCTCCCTGGTCTTCA	200	0.993	94.14	KX247392
*bactin*	F:TGGAGGTACCACCATGTACCC R:CACATCTGCTGGAAGGTGGA	167	0.998	96.42	
*rpl6*	F:GTGGTCAAACTCCGCAAGA R:GCCAGTAAGGAGGATGAGGA	149	0.995	90.06	Akbarzadeh et al. (35)
*gapdh*	F:ACACCCGCTCATCAATCTTT R:AGGTCCACGACTCTGTTGCT	114	0.997	91.13	
*rpl13*	F:GGACGTGGTTTCACCCTTG R:GGGAAGAGGATGAGTTTGGA	166	0.998	96.78	
*ef1a*	F:GGACTCCACTGAGCCACCT R:GGGTTGTAGCCGATCTTCTTG	89	0.989	108.07	
*ubq*	F:GGAAGACCATCACCCTGGA R:ACAGCGTGCGACCATCTT	140	0.997	87.33	

*foxl2, forkhead box L2; cyp19a1, cytochrome P450, family 19, subfamily a, member 1; ar, androgen receptor; era, estrogen receptor α; erb, estrogen receptor b; vtg, vitellogenin; vtgr, vitellogenin receptor; lpl, lipoprotein lipase; F, forward; R, reverse; Eff, efficiency*.

For gene expression analysis in ovary and liver *in vivo*, and in ovarian explants *in vitro*, the relative standard curve method was used to extrapolate relative expression of target genes (34). The standard curves were constructed from dilution series of pooled cDNA (including six dilutions from 1/5 to 1/5^6^). For genes in hepatic explants *in vitro*, internal reference method was used for quantitative real-time PCR. The reference gene *(b-actin, rpl6, gapdh, rpl13, ef1a, ubq*) was screened during the pre-experiment. Primers of six reference genes for RT-qPCR were taken from Akbarzadeh et al. (35). BestKeeper (37), Delta CT (38), and Normfinder (39) was used to evaluate the expression stability of each candidate reference gene.

Quantitative PCRs were run on an ABI PRISM 7500 Real-Time PCR System at a final reaction volume of 20 μL, containing 0.5 μL of 5-fold diluted cDNA template, 10 μL 2 × SYBR green real-time PCR master mix (ABI), 0.2 μL of each primer (10 μM) and 9.1 μL double-distilled H_2_O. The amplification protocol was as follows: 2 min at 50°C, 5 min at 95°C, 40 cycles of denaturation 30 s at 95°C, annealing 30 s at 60°C, and extension 30 s at 72°C, followed by dissociation curve analysis. Negative controls were prepared with sterile deionized water instead of cDNA template. All reactions were run in triplicate.

### Statistical Analyses

Statistical analyses were performed by SPSS Statistics 22.0. Data of fork length, body weights, liver weights, and gonad weights were showed as mean ± SD. Concentrations of sex steroids, Vtg, and real-time PCR data were expressed as mean ± SEM. All data were tested firstly for normality and homogeneity of variance using the Shapiro-Wilk test and the Levene's test, respectively. When the assumptions were met, data were analyzed by one-way analysis of variance (ANOVA) followed by the Tukey's test. If the data did not fit a normal distribution, they were log transformed for further study. When the data failed to meet these assumptions even after being transformed, the non-parametric test with Krusal-Wallis test was used on all pairwise comparisons. The difference of liver and gonad weights between groups was analyzed by ANCOVA with weight as covariate. All statistical analyses and comparisons were determined at *P* < 0.05 level (significant).

## Results

### Effects of 11-KT Treatment *in vivo*

#### Changes of Body Weight, Organ Weight, HSI, and GSI

**Figure 1:**
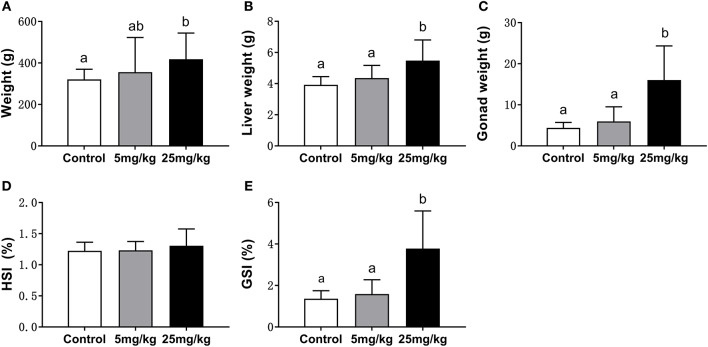
Comparison of body weight **(A)**, liver weight **(B)**, gonad weight **(C)**, HIS **(D)**, and GSI **(E)** among groups (mean ± SD, *n* = 18). Different lowercase letters denote significant differences (*P* < 0.05). ANOVA was used to analyze the significance of differences in weight, HSI and GSI, and ANCOVA was used to analyze the significance of differences in liver weight and gonad weight.

At the 30 day post-implantation time point, the average weight of the 25 mg/kg group (418.08 ± 51.54 g) was significantly higher than that in the control group (320.73 ± 19.99 g) (*P* = 0.018) and had no difference with the 5 mg/kg group (355.50 ± 68.35 g) (*P* = 0.156) (Figure 1A). With covariance analysis, the average liver and gonadal weights were higher in the 25 mg/kg group than those in the other two groups (*P* = 0.042 and *P* = 0.037, for liver and gonad, respectively; Figures 1B,C). There were no differences in HSI among groups (*P* = 0.187, Figure 1D). The GSI was higher in the 25 mg/kg group than that in the control (*P* = 0.008) and the 5 mg/kg groups (*P* = 0.022) (Figure 1E).

#### Histological Observation of Sterlet Ovary

**Figure 2:**
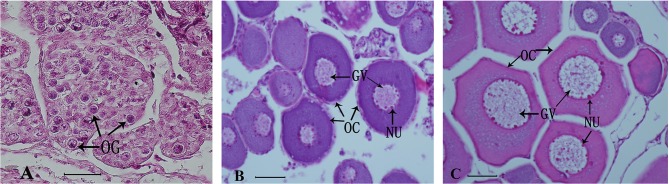
Representative histological observation of sterlet gonad following *in vivo* 11-KT treatment **(A)** ovary at Chromatin nucleolus stage (stage I, *n* = 9): oogonia and oocytes were under the ovigerous lamella in the invagination on the lateral side of the ovary. **(B,C)** Ovary at stage II (perinucleolus stage, *n* = 9): the ovigerous lamellae contain few clusters of oogonia and many enlarged follicles with perinucleolar oocytes and nucleoli were distributed around the inner part of the nuclear envelope. GV, germinal vesicle; NU, nucleolus; OC, oocyte; OG, oogonia. Scale bars = 50 μM. Magnification 400X.

The effect of treatment with 11-KT on ovarian development was determined based on histological observation. The control group was at Chromatin nucleolus stage (*n* = 6, stage I) (Figure 2A). No masculinization characteristics was found in the experimental groups. Half samples in the 5 mg/kg group were at stage I (*n* = 3, Figure 2A), while the other half were at the perinucleolus stage (stage II) (*n* = 3, Figure 2B). All samples in 25 mg/kg developed into stage II (*n* = 6, Figure 2C).

#### Gene Expression Profiles for *ar, era, erb, lpl*, and *Vtg* in Liver

**Figure 3:**
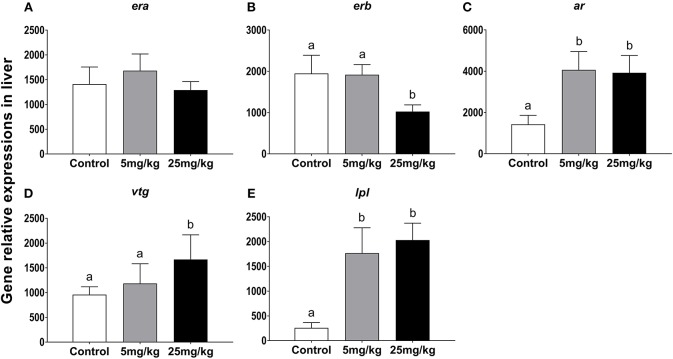
The effect of 11-KT implanting on the expression of gene *era* **(A)**, *erb* **(B)**, *ar* **(C)**, *vtg* **(D)** and *lpl* **(E)** in sterlet liver (mean ± SEM, *n* = 18). Different lowercase letters denote a significant difference (*P* < 0.05). ANOVA was used to analyze significance of differences among groups.

While mRNA levels for *era* were highest in the 5 mg/kg group, there were no differences among groups (*P* = 0.820, Figure 3A). Expression levels for *erb* were not different between the control and the 5 mg/kg groups (*P* = 0.978) but decreased in the 25 mg/kg group (*P* = 0.024, Figure 3B). The expression levels for *ar* were higher in both experimental groups as compared to the control group (*P* = 0.019, Figure 3C). *Vtg* expression increased with 25 mg/kg 11-KT treatment, as compared to both the control and 5 mg/mL groups, with no difference in expression between the latter groups (*P* = 0.043), but no significant difference was detected between control and the 5 mg/kg group (*P* = 0.302) (Figure 3D). Expression levels of *lpl* increased in both experimental groups with respect to the control (*P* < 0.001), with no differences between them (*P* = 0.715, Figure 3E).

#### Gene Expression Profiles for *foxl2, cyp19a1, era, erb, ar, Vtgr* in Ovary

**Figure 4:**
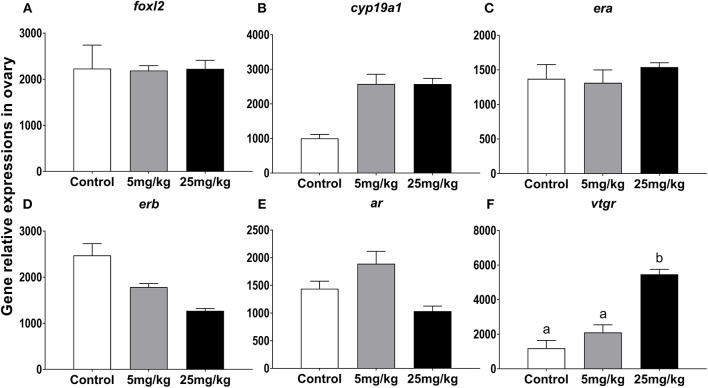
The effect of 11-KT implanting on the expression of gene *foxl2* **(A)**, *cyp19a1* **(B)**, *era* **(C)**, *erb* **(D)**, *ar* **(E)**, and *vtgr* **(F)** in sterlet ovary (mean ± SEM, *n* = 18). Different lowercase letters denote a significant difference (*P* < 0.05). ANOVA was used to analyze significance of differences among groups, except that *foxl2, erb*, and *vtgr* were analyzed with a non-parametric test followed by Krusal-Wallis test.

In ovary, 11-KT treatment increased *vtgr* (vitellogenin receptor) expression only at 25 mg/kg (*P* = 0.04 and *P* = 0.02, compared to 5 mg/kg and control groups, respectively; Figure 4F). There were no significant expression changes detected for each of the other transcripts. However, it's worth noting that the expression of *cyp19a1* had a growing trend in both experimental groups (*P* = 0.058, Figure 4B) and *erb* showed a downward trend with increasing concentrations of 11-KT (*P* = 0.052, Figure 4D).

#### Changes in T, E2, and Vtg Concentrations in Serum

**Figure 5:**
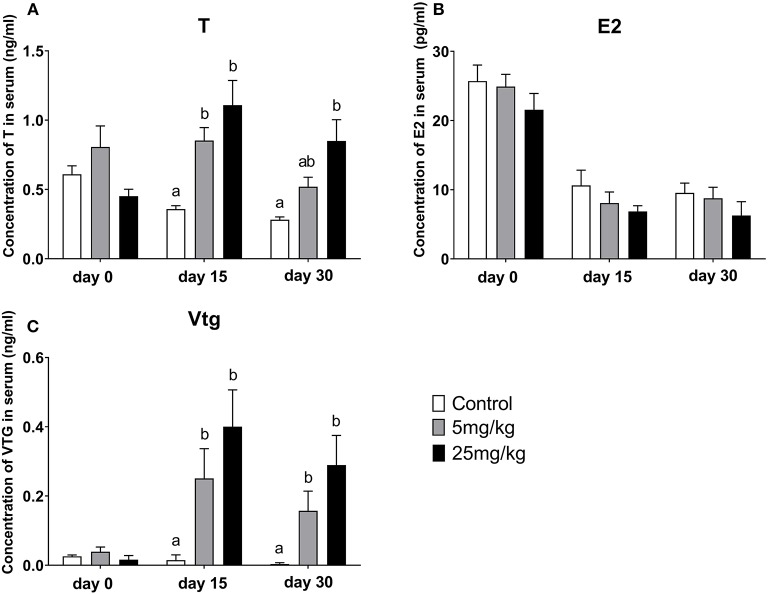
Change of serum T **(A)**, E2 **(B)**, and Vtg **(C)** concentrations during the 11-KT silastic strips implanting (mean ± SEM, *n* = 18). Different lowercase letters denote a significant difference (*P* < 0.05). ANOVA was used to analyze the significance of differences between groups, with the exception of Vtg, which on days 15 and 30 was analyzed by a non-parametric test followed by Kruskal-Wallis.

Before implantation (day 0), T, E2, and Vtg concentrations in serum were similar among groups (Figure 5). Following implantation for 15 days and compared to the control group (0.35 ± 0.02 ng/mL), T concentrations increased in both the 5 mg/kg group (0.85 ± 0.09 ng/mL, *P* = 0.001) and the 25 mg/kg group (0.40 ± 0.11 ng/mL, P ≤ 0.001). However, there was no difference between the experimental groups (*P* = 0.492) (Figure 5A). A similar trend was observed for Vtg concentrations with differences between the control group (0.01± 0.01 ng/mL), the 5 mg/kg groups (0.25 ± 0.08 ng/mL) and the 25 mg/kg (0.40 ± 0.11 ng/mL) (*P* = 0.001, Figure 5C). However, E2 concentrations showed a downward trend with 11-KT treatment, although differences among groups were not significant (*P* = 0.255, Figure 5B). After 30 days implantation, T concentrations showed an increasing trend but were only different between the control group (0.28 ± 0.02 ng/mL) and the 25 mg/kg group (0.85 ± 0.15 ng/mL, *P* = 0.007) and not with the 5 mg/kg group (0.52 ± 0.07 ng/mL, *P* = 0.126) (Figure 5A). Results were similar for Vtg: in both the 5 mg/kg (0.15 ± 0.05 ng/mL) (*P* = 0.028) and the 25 mg/kg (0.29 ± 0.08 ng/mL) groups, concentrations were higher (*P* = 0.003) than those in the control group (0.003 ± 0.003 ng/mL) (Figure 5C). E2 levels showed a decreasing trend (9.54 ± 1.42, 8.77±1.59, 6.275 ± 2.00 ng/mL, respectively) with no significant difference among groups (*P* = 0.188, Figure 5B).

### Effects of 11-KT Treatment *in vitro*

#### Histological Observation of Ovary

**Figure 6:**
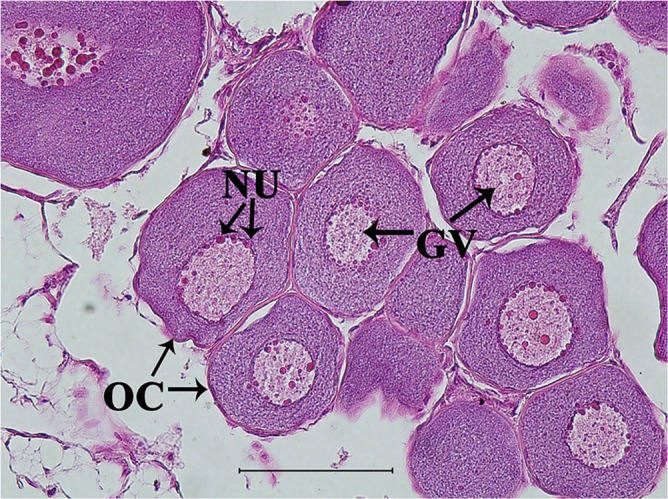
Representative histological observation of sterlet gonad following *in vitro* 11-KT treatment. Ovary at stage II: oocyte development with number and volume increasing, nucleolus and germinal vesicle presence. GV, germinal vesicle; NU, nucleolus; OC, oocyte. Scale bars is 100 μm. Magnification 400X.

The ovarian development of samples *in vitro* was confirmed by histological observation and results showed all samples developed into stage II (Figure 6).

#### Gene Expression Profiles of *era, erb, ar, lpl, cyp19a1, Vtg* in Hepatic Explant

**Figure 7:**
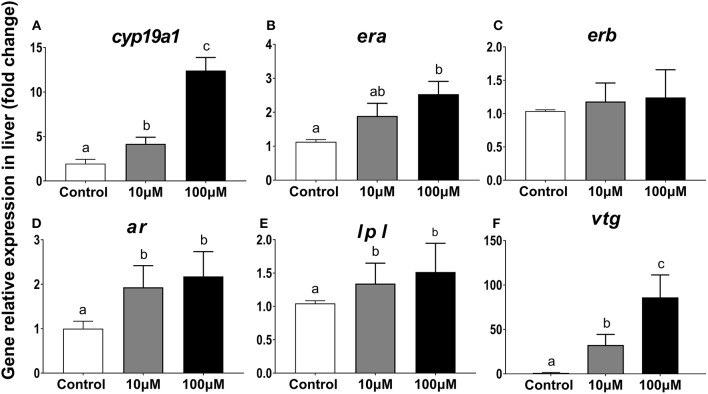
Effects of 11-KT on gene expression of *cyp19a1* **(A)**, *era* **(B)**, *erb* **(C)**, *ar* **(D)**, *lpl* **(E)**, and *vtg* **(F)** in hepatic explant cultured with 11-KT (10 and 100 μM) or without 11-KT *in vitro* (mean ± SEM, *n* = 3). Expression for test genes was compared against that for the reference gene β*-actin*. Different lowercase letters denote a significant difference (*P* < 0.05). ANOVA was used to analyze significance of differences among groups, except for *era* and *erb*, which were analyzed with a non-parametric test followed by the Krusal-Wallis test.

From the reference primers screened, b*-actin* was best suited (*SD* = 0.22, in BestKeeper; *SD* = 0.57 in Delta CT; *SD*= 0.052 in Normfinder; *P* < 0.05, Table 1). For *cyp19a1*, expression in the 100 μm group was 6.4 times higher than that in the control group (*P* = 0.001) and 3 times higher than that in the 10 μM group (*P* = 0.002) (Figure 7A). For *era*, expression in the 100 μm group was 2.2 times higher than that in the control group (*P* = 0.033) and 1.3 times higher than that in the 10 μM group (*P* = 0.108) (Figure 7B). Expression for *erb* increased slightly with increasing 11-KT concentrations but there were no differences showed among groups (*P* = 0.838, Figure 7C). Expression for *ar* and *lpl* in the experimental groups were higher than those in the corresponding control group (*P* = 0.017; *P* ≤ 0.001, respectively) but no significant differences were detected between the two experimental groups (*P* = 0.784; *P* = 0.999, respectively) (Figures 7D,E). *Ar* expression was 1.93 times higher in the 10 μM group (*P* = 0.017) and 2.18 times higher in the 100 μM group (*P* = 0.019) than that in the control group (Figure 7D). The expression of *lpl* was 1.3 times higher in the 10 μM group (*P* = 0.001), and 1.5 times higher in the 100 μM group (*P* ≤ 0.001) than that in the control group (Figure 7E). Notably, 11-KT had a marked effect on *vtg* expression. In the 100 μM group, it was 30 times higher than that in the 10 μM group (*P* = 0.002), and 80 times higher than that in the control group (*P* = 0.01) (Figure 7F).

#### Changes in T, E2, and Vtg Concentrations in the Haptic Explants Culture Medium

**Figure 8:**
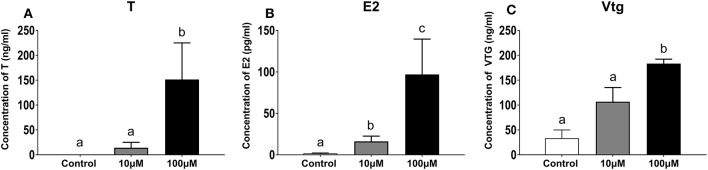
Concentration of T **(A)**, E2 **(B)**, and Vtg **(C)** in hepatic explant cultured with 11-KT (10 and 100 μM) or without 11-KT *in vitro* (mean ± SEM, *n* = 3). Different lowercase letters denote a significant difference (*P* < 0.05). ANOVA was used to analyze the significance of differences between groups, except that T and Vtg were analyzed with non-parametric tests followed by the Krusal-Wallis test.

Concentrations of T and E2 in the following 5 days of liver explant culture increased significantly for culture medium supplemented with 100 μM 11-KT. T concentrations were higher in the 100 μM group (151.30 ± 73.50 ng/mL) than both those in 10 μM groups (14.14 ± 10.90 ng/mL) (*P* = 0.058) and the control group (0.01 ± 0.00 ng/mL) (*P* = 0.026) (Figure 8A). Concentrations of E2 in the 100 μM group (96.88 ± 42.63 pg/mL) was 6 times more than that in the 10 μM group (16.61 + 6.40 pg/mL) (*P* = 0.002), and 60 times more than that in the control group (1.59 + 0.69 pg/mL) (*P* = 0.001) (Figure 8B). Vtg showed an upward trend with increasing 11-KT concentrations (*P* = 0.035). The concentration in the 100 μM (0.18 ± 0.00 ng/mL) was 1.8 times as high as that of the 10 μM (0.10 ± 0.02 ng/mL) (*P* = 0.026), and 6 times as high as that in the control group (0.03 ± 0.01 ng/mL) (Figure 8C).

#### Gene Expression Profiles for *foxl2, cyp19a1, era, erb, ar, Vtgr* in Ovarian Explants

**Figure 9:**
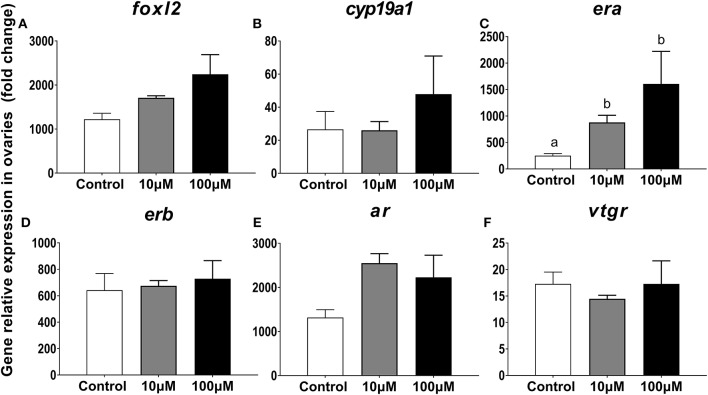
Effects of 11-KT on gene expression of *foxl2* **(A)**, *cyp19a1* **(B)**, *era* **(C)**, *erb* **(D)**, *ar* **(E)**, and *vtgr* **(F)** in ovarian explant cultured with 11-KT (10 and 100 μM) or without 11-KT *in vitro* (mean ± SEM, *n* = 3). Different lowercase letters denote a significant difference (*P* < 0.05). ANOVA was used to analyze significance of differences among groups.

After incubation of ovarian explants in a culture medium with or without 11-KT for 5 days, changes in expression levels were only observed for *era* in both experimental groups as compared to the control group (*P* = 0.005, Figure 9C). The expression of other genes *foxl2, cyp19a1*, and *erb* displayed a rising trend with increasing 11-KT concentration, but there was no significant difference between groups (*P* = 0.095, Figure 9A; *P* = 0.214, Figure 9B; *P* = 0.838, Figure 9D, respectively). The *ar* expression levels were the highest in the 10 μM group followed by the 100 μM group without detection of significant difference (*P* = 0.052, Figure 9E). Expression of *Vtgr* decreased slightly in the 10 μM group with no differences observed among groups (*P* = 0.777, Figure 9F).

#### Changes in T and E2 Concentrations in the Ovarian Explants Culture Medium

**Figure 10:**
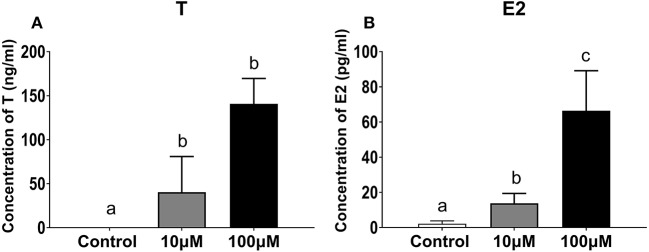
Concentration of T **(A)** and E2 **(B)** in ovarian explant culture medium with 11-KT (10 and 100 μM) or without 11-KT *in vitro* (mean ± SEM, *n* = 3). Different lowercase letters denote a significant difference (*P* < 0.05). ANOVA was used to analyze the significance of differences between groups.

Both T and E2 concentrations increased in the ovarian tissue culture medium with 11-KT supplementation. Concentrations of T were higher in the experimental groups than in the control group (0.02 ± 0.01 ng/mL, *P* = 0.001), with no significant differences between the 10 μM group (140.75 ± 28.87 ng/mL) and the 100 μM group (40.27 ± 40.54 ng/mL) (*P* = 0.058, Figure 10A). E2 concentrations were 4.8 and 33 times higher in the 100 μM group (66.36 ± 22.79 pg/mL) than in the 10 μM group (13.77 ± 5.63 pg/mL; *P* = 0.024) and the control group (2.17 ± 1.63 pg/mL; *P* = 0.002) (Figure 10B).

## Discussion

Many studies have shown that *foxl2* and *cyp19a1* are closely related to sex differentiation, as well as ovarian development and maintenance (40–42), such as in mammalian (43), avian (44), and teleost (42, 45, 46) females, but not during male development. There were similar results in the sturgeon, like lake sturgeon (47), shortnose sturgeon (48, 49), Russian sturgeon (50), and sterlet (34, 51). Moreover, the adult female zebrafish could be sex-reversed to male by inhibiting aromatase (52). Treatment with exogenous androgens may result in female fish masculinization, or even sex-reversed to male (53, 54). For instance, the sex ratio of juvenile paddlefish (*Polyodon spathula*) could be reversed by implanting MT (17α-methyltestosterone) silica stripes (55). The effects of MT treatment depend upon the dosage and timing. Hence, oral supplementation of hybrid sturgeon (bester) with MT at 25 mg/kg MT failed to induce masculinization for 14–31 months old females, while at 1 mg/kg it successfully induced feminization or masculinization in 3–18 months old undifferentiated bester (56). In this study, exogenous 11-KT treatment had no effect on the expression of *foxl2* and *cyp19a1*, and did not induce ovarian masculinization or sex reversal. Similar results were detected from the 11-KT implantation on short-finned eel (57). Notably, the different effects between MT and 11-KT may stem from their susceptibility to be aromatized. MT can be aromatized to ME2 (58), which acts as an exogenous estrogen, while 11-KT cannot (54, 58). Therefore, these studies suggest that the enzymes governing hormone synthesis may be more important in regulating sexual differentiation than the sex hormones themselves (59–61). Gene *foxl2, cyp19a1*, and receptors showed an upward trend of expression with no significant differences *in vitro* in this study, indicating that the ovarian response to 11-KT was a passive process which only regulated by E2 and the expression of *era*.

11-KT plays an important role in the growth of previtellogenic oocyte in few fish. 11-KT induces oocyte growth in previtellogenic captive beluga *in vivo* (62). In previtellogenic female short-finned eels, 11-KT implanting induced maturation changes including “chisel-shaped” snouts, enlarged eyes, increased liver weight, and hastened development of oocytes from stage II to III (18). *In vitro*, 11-KT played an important role in Vldl transfer and accumulation in Japanese eel (19), and increasing oocyte size, lipid droplet surface area, as well as in the number of oil droplets in the New Zealand long-finned eel (21). 11-KT also induced a significant increase in the growth of previtellogenic oocytes and their nuclei in a dose-dependent manner in short-finned eels *in vitro* (63). Studies of both *in vitro* and *in vivo* with 11-KT treatment hastened the growth and development of previtellogenic oocytes and suggested a role for androgen in controlled early oocyte growth in the Atlantic cod (23, 24). In addition, late perinucleolar-stage follicles in Coho Salmon increased significantly in size after 7 days of 11-KT treatment *in vitro* (22). Conversely, 11-KT treatment *in vivo* in previtellogenic captive hapuku (*Polyprion oxygeneios*) had no effects on ovarian development and hepatic total lipid concentrations (64). These results suggest that 11-KT promotes ovarian development in only a few fishes. In this study, no significant changes in morphological traits were observed in sterlet, while liver weight, gonad weight, and GSI significantly increased in this study. Thus, it can be inferred that implantation of exogenous 11-KT promotes early ovarian development of sterlet, and the effects have a dose-dependent manner.

In the process of development, fish oocytes store fat as a source of energy for larvae development (65). In the early stages of vitellogenesis, ovaries secret estrogen (mainly E2) to stimulate lipids and Vtg synthesis in the liver through the estrogen receptor pathway (66). Then these synthetic products bind to very low density lipoproteins (Vldl) and other carriers, to be transported via the bloodstream to developing oocytes (65, 67). Meanwhile, the corresponding transport proteins in the ovary, Vtgr and Vldl, were responsible for lipids and Vtg absorption through receptor-mediated endocytosis and enzyme hydrolysis (68). *Vtg* expression increased *in vivo* following serum Vtg increasing in this study. The expression of *vtgr* in the ovary, which mediates Vtg binding and transport, increased significantly. The *lpl* expression, which is closely related to lipid synthesis, also increased significantly. In the ovary of short-finned eel *lpl* expression was also significantly increased by the effects of 11-KT both *in vivo* and *in vitro* (69). Some studies have shown that androgens could stimulate the *vtg* expression or Vtg synthesis in the liver cells. Different concentrations of T, androsterone, and MT-induced *vtg* expression during *in vitro* culture of hepatocytes from immature male rainbow trout (*Oncorhynchus mykiss*) (70). Dihydrotestosterone (DHT), T, and MT could stimulate Vtg synthesis in previtellogenic liver cells of tilapia (*Oreochromis mossambicus*) (71). In Japanese eel, culture of previtellogenic ovarian fragments with 11-KT and Vldl increased oocyte size and lipid droplet surface area (20). In this study, treatment with 11-KT also increased the expression of *lpl* and *vtg* in liver explants with Vtg concentrations increasing. Altogether, both *in vivo* and *in vitro* results support that 11-KT not only induce Vtg and lipid synthesis, but also may play an important role in Vtg and lipid transport and absorption.

The *ar* gene, which is expressed in the ovary of teleost fish (63, 72–75), is localized in ovarian follicle cells with highest expression during the oil droplet accumulation phase in oocytes (76). 11-KT treatment increased the size of late perinucleolar-stage follicles, but an Ar antagonist (flutamide) restrained this growth promoting effect in silver salmon (22). Er subtypes in different fish appeared to have non-redundant roles (77, 78). Both *era* and *erb* in the sturgeon had a similar domain to those in other fish (79), and had a strong affinity to E2 (79, 80). Gene expression of *ar* increased in liver *in vivo*, while *era* expression did not change, and *erb* expression decreased in this study. In the ovary, 11-KT had no effect on the expression of the sex steroid receptors (*ar, era, erb*). Therefore, it was speculated that *in vivo* in sterlet, extraneous androgen 11-KT may act directly through the Ar pathway in the liver. There may be indirect pathway to effects on the Ar in the ovary. These assumptions require further investigation. In *in vitro* experiment, it is very helpful to explore the effects of various external factors on the regulation of gametogenesis, independent from serum and inter-tissue interactions (20). It was inferred that Ar antagonist flutamide reduced androgen-stimulated Vtg synthesis, indicating that this process was directly regulated via Ar (71). In our study *in vitro, ar* expression increased in hepatic explants, consistent with the *in vivo* study. *Era* increased in the experimental groups while *erb* showed a slight upward trend. These results indicate *era* is more sensitively for responsible to 11-KT treatment than *erb* in the early development of oocytes. However, results on goldfish (*Carassius auratus*) disagreed with ours in that the increase of E2 promoted the expression of *erb* in the liver, and the latter directly promoted the synthesis of Vtg (81). Therefore, the function of Era in previtellogenic sterlets requires further study.

The process by which T can be converted to 11-KT is at downstream in steroidogenesis, and is not reversible (25). It was found 11-KT could be converted to 11-keto-5a-dihydrotestosterone by human steroid 5a-reductase type 1 (SRD5A1) (82) or to 11-ketoandrostenedione by human 17b-HSD (17b-hydroxysteroid dehydrogenase) (83) in the studies of androgen dependent diseases. However, similar results were not found in other species and need further study in sturgeon. The balance between androgens and estrogens is critical during fish development (84). *In vivo*, serum T increased with 11-KT treatment. It is possible that a too exogenous 11-KT breaks the sex steroid hormone balance and converts to other androgen to maintain sex steroid homeostasis. On the other hand, a positive effect of ovarian development was supposed to be the elevation of testosterone. These assumptions still needed to be proved. Conversely, E2 concentrations in serum showed a downward trend (Figure 5B), implying that paracrine system synthesized E2 to promote the synthesis of vtg. This was consistent with the results of 11-KT implantation in short-finned eels (57). Conversely, the concentrations of T and E2 in both hepatic and ovarian culture medium was increased, suggesting high level 11-KT could promote the synthesis of T and E2 in both tissues. The discrepancy in E2 concentrations between *in vivo* and *in vitro* suggests the existence of a compensatory mechanism in the liver. Therefore, it hypothesizes that when the exogenous 11-KT increased, followed by insufficient ovarian E2 synthesis, the liver would compensate by synthesizing E2 to maintain the normal ovarian development and sex steroid hormone homeostasis, as well as to promote Vtg synthesis.

In summary, we report that 11-KT induced ovarian development without ovarian masculinization or sex reversal *in vivo*, as well as Vtg and lipid synthesis *in vivo* and *in vitro*. To our knowledge, this is the first report in sturgeon to describe the 11-KT effect on the development of the previtellogenic sterlet. Through detection of T, E2, and 11-KT concentrations in gonadal development stages of breeding Siberia sturgeon during a natural breeding season, Hamlin et al. (30) reported 11-KT in females increased, beginning at the previtellogenic stage and peaking at the germinal vesicle stage, with a concomitant increase in E2 concentrations. Serum 11-KT concentrations were low in previtellogenic females of the Amur sturgeon (*A. schrenckii*), but increased at the beginning of vitellogenesis and peaked before E2 concentrations (31). Therefore, it appears that in sturgeon, 11-KT is an important factor initiating Vtg synthesis at previtellogenic stage, potentially through the activation of E2 secretion via Ar and Era signal pathways. However, detailed understanding of these pathways requires additional studies, such as RNA-sequencing or microRNA regulation, to decipher the molecular mechanisms involved.

## Data Availability Statement

All datasets generated for this study are included in the article/supplementary material.

## Ethics Statement

The animal study was reviewed and approved by Academic Committee of Beijing Fisheries Research Institute.

## Author Contributions

WW and HH conceived and designed the experiments. WW, ZT, AS, YD, and TD performed the experiments. WW and HH analyzed the data and interpreted the results. WW, HH, ZT, and HZ contributed reagents or materials or analysis tools. WW and HH wrote and revised the paper.

### Conflict of Interest

The authors declare that the research was conducted in the absence of any commercial or financial relationships that could be construed as a potential conflict of interest.

## References

[1] Polzonetti-Magni AM Mosconi G Soverchia L Kikuyama S Carnevali O. Multihormonal control of vitellogenesis in lower vertebrates. Int Rev Cytol. (2004) 239:1–46. 10.1016/S0074-7696(04)39001-7 15464851

[2] Patiño R Sullivan CV Ovarian follicle growth, maturation, and ovulation in teleost fish. Fish Physiol Biochem. (2002) 26:57–70. 10.1023/A:1023311613987

[3] Nagahama Y The functional morphology of teleost gonads. Fish Physiol. (1983) 9:223–75. 10.1016/S1546-5098(08)60290-3

[4] Kime DE ‘Classical’ and ‘non-classical’ reproductive steroids in fish. Rev Fish Biol Fish. (1993) 3:160–80. 10.1007/BF00045230

[5] Doroshov SI Moberg GP Eenennaam JPV Observations on the reproductive cycle of cultures white sturgeon, *Acipenser transmontanus*. Environ Biol Fishes. (1997) 48:265–78. 10.1023/A:1007336802423

[6] Webb MAH Eenennaam JPV Doroshov SI Moberg GP Preliminary observations on the effects of holding temperature on reproductive performance of female white sturgeon, *Acipenser transmontanus* Richardson. Aquaculture. (1999) 176:315–29. 10.1016/S0044-8486(99)00108-8

[7] Webb MA Feist GW Trant JM Van Eenennaam JP Fitzpatrick MS Schreck CB. Ovarian steroidogenesis in white sturgeon (*Acipenser transmontanus*) during oocyte maturation and induced ovulation. Gen Comp Endocrinol. (2002) 129:27–38. 10.1016/S0016-6480(02)00508-7 12409093

[8] Malekzadeh Viayeh R Webb M Hallajian A Kazemi R Pahlavan Yali M Biochemical and morphometric parameters as indicators of sex and gonadal stages in maturing persian sturgeon, *Acipenser persicus*. J Appl Ichthyol. (2006) 22:364–8. 10.1111/j.1439-0426.2007.00986.x

[9] Amiri BM Maebayashi M Hara A Adachi S Yamauchi K Ovarian development and serum sex steroid and vitellogenin profiles in the female cultured sturgeon hybrid, the bester. J Fish Biol. (1996) 48:1164–78. 10.1111/j.1095-8649.1996.tb01812.x

[10] Miura T Yamauchi K Takahashi H Nagahama Y. Hormonal induction of all stages of spermatogenesis *in vitro* in the male Japanese eel (*Anguilla japonica*). Proc Natl Acad Sci USA. (1991) 88:5774–8. 10.1073/pnas.88.13.5774 2062857 PMC51960

[11] Jianga JQ Kobayashi T Ge W Kobayashi H Tanaka M Okamoto M Fish testicular 11beta-hydroxylase: cDNA cloning and mRNA expression during spermatogenesis. FEBS Lett. (1996) 397:250–2. 10.1016/S0014-5793(96)01187-8 8955357

[12] Gill A Jamnongjit M Hammes SR. Androgens promote maturation and signaling in mouse oocytes independent of transcription: a release of inhibition model for mammalian oocyte meiosis. Mol Endocrinol. (2004) 18:97–104. 10.1210/me.2003-0326 14576339

[13] Hammes SR Steroids and oocyte maturation-a new look at an old story. Mol Endocrinol. (2004) 18:769–75. 10.1210/me.2003-0317 14630999

[14] Truscott B So YP Nagler JJ Idler DR. Steroids involved with final oocyte maturation in the winter flounder. J Steroid Biochem Mol Biol. (1992) 42:351–6. 10.1016/0960-0760(92)90139-A 1606046

[15] Mylonas C Magnus Y Klebanov Y Gissis A Zohar Y Reproductive biology and endocrine regulation of final oocyte maturation of captive white bass. J Fish Biol. (1997) 51:234–50. 10.1111/j.1095-8649.1997.tb01662.x

[16] Kazeto Y Tosaka R Matsubara H Ijiri S Adachi S. Ovarian steroidogenesis and the role of sex steroid hormones on ovarian growth and maturation of the Japanese eel. J Steroid Biochem Mol Biol. (2011) 127:149–54. 10.1016/j.jsbmb.2011.03.013 21414407

[17] Lokman PM Harris B Kusakabe M Kime DE Schulz RW Adachi S. 11-Oxygenated androgens in female teleosts: prevalence, abundance, and life history implications. Gen Comp Endocrinol. (2002) 129:1–12. 10.1016/S0016-6480(02)00562-2 12409090

[18] Rohr DH Lokman PM Davie PS Young G. 11-Ketotestosterone induces silvering-related changes in immature female short-finned eels, *Anguilla australis*. Comp Biochem Physiol A Mol Integr Physiol. (2001) 130:701–14. 10.1016/S1095-6433(01)00402-0 11691606

[19] Endo T Todo T LokMan PM IjIrI S Adachi S Yamauchi K *In vitro* induction of oil droplet accumulation into previtellogenic oocytes of Japanese eel, *Anguilla japonica*. Cybium. (2008) 32:239–40.

[20] Endo T Todo T Lokman PM Kudo H Ijiri S Adachi S. Androgens and very low density lipoprotein are essential for the growth of previtellogenic oocytes from Japanese eel, *Anguilla japonica, in vitro*. Biol Reprod. (2011) 84:816–25. 10.1095/biolreprod.110.087163 21148104

[21] Matsubara M Lokman P Senaha A Kazeto Y Ijiri S Kambegawa A Synthesis and possible function of 11-ketotestosterone during oogenesis in eel (*Anguilla spp*.). Fish Physiol Biochem. (2003) 28:353–4. 10.1023/B:FISH.0000030585.22093.7a

[22] Forsgren K Young G. Stage-specific effects of androgens and estradiol-17beta on the development of late primary and early secondary ovarian follicles of coho salmon (*Oncorhynchus kisutch*) *in vitro*. Biol Reprod. (2012) 87:1166–70. 10.1095/biolreprod.111.098772 22674392

[23] Kortner TM Rocha E Arukwe A. Androgenic modulation of early growth of Atlantic cod (*Gadus morhua* L.) previtellogenic oocytes and zona radiata-related genes. J Toxicol Environ Health A. (2009) 72:184–95. 10.1080/15287390802539020 19184733

[24] Kortner TM Rocha E Arukwe A. Previtellogenic oocyte growth and transcriptional changes of steroidogenic enzyme genes in immature female Atlantic cod (*Gadus morhua* L.) after exposure to the androgens 11-ketotestosterone and testosterone. Comp Biochem Physiol A Mol Integr Physiol. (2009) 152:304–13. 10.1016/j.cbpa.2008.11.001 19036348

[25] Borg B Androgens in teleost fishes. Comp Biochem Physiol C Toxicol Pharmacol. (1994) 109:219–45. 10.1016/0742-8413(94)00063-G

[26] Cuisset B Fostier A Williot P Bennetau-Pelissero C Le Menn F. Occurrence and *in vitro* biosynthesis of 11-ketotestosterone in siberian sturgeon, *Acipenser baeri* brandt maturing females. Fish Physiol Biochem. (1995) 14:313–22. 10.1007/BF00004069 24197499

[27] Barannikova IA Bayunova LV Semenkova TB Serum levels of testosterone, 11-ketotestosterone and oestradiol-17β in three species of sturgeon during gonadal development and final maturation induced by hormonal treatment. J Fish Biol. (2004) 64:1330–8. 10.1111/j.0022-1112.2004.00395.x

[28] Ceapa C Williot P Le Menn F Davail-Cuisset B Plasma sex steroids and vitellogenin levels in stellate sturgeon (*Acipenser stellatus* Pallas) during spawning migration in the Danube River. J Appl Ichthyol. (2002) 18:391–6. 10.1046/j.1439-0426.2002.00370.x

[29] Cuisset B Rouault T Williot P Estradiol, testosterone, 11-ketotestosterone, 17, 20β-dihydroxy-4-pregnen-3-one and vitellogenin plasma levels in females of captive European sturgeon, *Acipenser sturio*. J Appl Ichthyol. (2011) 27:666–72. 10.1111/j.1439-0426.2011.01730.x

[30] Hamlin HJ Milnes MR Beaulaton CM Albergotti LC Guillette LJ Gonadal stage and sex steroid correlations in siberian sturgeon, *Acipenser baeri*, habituated to a semitropical environment. J World Aquacult Soc. (2011) 42:313–20. 10.1111/j.1749-7345.2011.00469.x

[31] Hu HX Reproductive Ednocrine and Artificial Progation Regulation in Cultured Amur Sturgeon, Acipenser Schrenckii. Guangzhou: Sun Yat-sen University, Life Science Academy (2006).

[32] Billard R Lecointre G Biology and conservation of sturgeon and paddlefish. Rev Fish Biol Fish. (2001) 10:355–92. 10.1023/A:1020331818648

[33] Simpson TH Wright RS. A radioimmunoassay for 11-oxotestosterone: its application in the measurement of levels in blood serum of rainbow trout (*S. Gairdneri*). Steroids. (1977) 29:383–98. 10.1016/0039-128X(77)90007-1 871021

[34] Wang W Zhu H Dong Y Tian Z Dong T Hu H. Dimorphic expression of sex-related genes in different gonadal development stages of sterlet, *Acipenser ruthenus*, a primitive fish species. Fish Physiol Biochem. (2017) 43:1557–69. 10.1007/s10695-017-0392-x 28963671

[35] Akbarzadeh A Farahmand H Mahjoubi F Nematollahi MA Leskinen P Rytkönen K. The transcription of l-gulono-gamma-lactone oxidase, a key enzyme for biosynthesis of ascorbate, during development of persian sturgeon *Acipenser persicus*. Comp Biochem Physiol B Biochem Mol Biol. (2011) 158:282–8. 10.1016/j.cbpb.2010.12.005 21199677

[36] Pfaffl MW. A new mathematical model for relative quantification in real-time RT–PCR. Nucleic Acids Res. (2001) 29:2002–7. 10.1093/nar/29.9.e45 11328886 PMC55695

[37] Pfaffl MW Tichopad A Prgomet C Neuvians TP. Determination of stable housekeeping genes, differentially regulated target genes and sample integrity: bestkeeper-excel-based tool using pair-wise correlations. Biotechnol Lett. (2004) 26:509–15. 10.1023/B:BILE.0000019559.84305.47 15127793

[38] Silver N Best S Jiang J Thein SL. Selection of housekeeping genes for gene expression studies in human reticulocytes using real-time PCR. BMC Mol Biol. (2006) 7:33. 10.1186/1471-2199-7-33 17026756 PMC1609175

[39] Andersen CL Jensen JL Orntoft TF. Normalization of real-time quantitative reverse transcription-PCR data: a model-based variance estimation approach to identify genes suited for normalization, applied to bladder and colon cancer data sets. Cancer Res. (2004) 64:5245–50. 10.1158/0008-5472.CAN-04-0496 15289330

[40] Piferrer F Guiguen Y Fish gonadogenesis. Part II: molecular biology and genomics of sex differentiation. Rev Fish Sci. (2008) 16:35–55. 10.1080/10641260802324644

[41] Sridevi P Chaitanya RK Dutta-Gupta A Senthilkumaran B. FTZ-F1 and FOXL2 up-regulate catfish brain aromatase gene transcription by specific binding to the promoter motifs. Biochim Biophys Acta. (2012) 1819:57–66. 10.1016/j.bbagrm.2011.10.003 22019437

[42] Wang DS Kobayashi T Zhou LY Paul-Prasanth B Ijiri S Sakai F. Foxl2 up-regulates aromatase gene transcription in a female-specific manner by binding to the promoter as well as interacting with ad4 binding protein/steroidogenic factor 1. Mol Endocrinol. (2007) 21:712–25. 10.1210/me.2006-0248 17192407

[43] Baron D Batista F Chaffaux S Cocquet J Cotinot C Cribiu E. Foxl2 gene and the development of the ovary: a story about goat, mouse, fish and woman. Reprod Nutr Dev. (2005) 45:377–82. 10.1051/rnd:2005028 15982462

[44] Govoroun MS Pannetier M Pailhoux E Cocquet J Brillard JP Couty I. Isolation of chicken homolog of the FOXL2 gene and comparison of its expression patterns with those of aromatase during ovarian development. Dev Dyn. (2004) 231:859–70. 10.1002/dvdy.20189 15517586

[45] Baron D Cocquet J Xia X Fellous M Guiguen Y Veitia RA. An evolutionary and functional analysis of FoxL2 in rainbow trout gonad differentiation. J Mol Endocrinol. (2004) 33:705–15. 10.1677/jme.1.01566 15591029

[46] Alam MA Komuro H Bhandari RK Nakamura S Soyano K Nakamura M. Immunohistochemical evidence identifying the site of androgen production in the ovary of the protogynous grouper *Epinephelus merra*. Cell Tissue Res. (2005) 320:323–9. 10.1007/s00441-004-1037-9 15778855

[47] Hale MC Jackson JR DeWoody JA. Discovery and evaluation of candidate sex-determining genes and xenobiotics in the gonads of lake sturgeon (*Acipenser fulvescens*). Genetica. (2010) 138:745–56. 10.1007/s10709-010-9455-y 20386959

[48] Amberg JJ Goforth RR Sepúlveda MS. Antagonists to the Wnt cascade exhibit sex-specific expression in gonads of sexually mature shovelnose sturgeon. Sex Dev. (2013) 7:308–15. 10.1159/000354280 23988442

[49] Amberg JJ Goforth RR Stefanavage T Sepúlveda MS. Sexually dimorphic gene expression in the gonad and liver of shovelnose sturgeon (*Scaphirhynchus platorynchus*). Fish Physiol Biochem. (2010) 36:923–32. 10.1007/s10695-009-9369-8 19941163

[50] Hagihara S Yamashita R Yamamoto S Ijiri S Adachi S Identification of genes involved in gonadal sex differentiation and the dimorphic expression pattern in undifferentiated gonads of Russian sturgeon *Acipenser gueldenstaedtii*. J Appl Ichthyol. (2014) 30:1557–64. 10.1111/jai.12588

[51] Wang W Zhu H Dong Y Dong T Tian Z Hu H Identification and dimorphic expression of sex-related genes during gonadal differentiation in sterlet *Acipenser ruthenus*, a primitive fish species. Aquaculture. (2019) 500:178–87. 10.1016/j.aquaculture.2018.10.001

[52] von Hofsten J Olsson PE. Zebrafish sex determination and differentiation: involvement of FTZ-F1 genes. Reprod Biol Endocrinol. (2005) 3:63–74. 10.1186/1477-7827-3-63 16281973 PMC1298332

[53] Wartman CA Hogan NS Hewitt LM McMaster ME Landman MJ Taylor S. Androgenic effects of a Canadian bleached kraft pulp and paper effluent as assessed using threespine stickleback (*Gasterosteus aculeatus*). Aquat Toxicol. (2009) 92:131–9. 10.1016/j.aquatox.2009.02.003 19261340

[54] Li GL Liu XC Lin HR. Effects of aromatizable and nonaromatizable androgens on the sex inversion of red-spotted grouper (*Epinephelus akaara*). Fish Physiol Biochem. (2006) 32:25–33. 10.1007/s10695-005-4900-z 20035475 PMC2780618

[55] Mims S Shelton W Clark J Steroid-induced sex reversal of paddlefish. In: Goetz E, Thomas P, editor. Proceedings of the 5th International Symposium on Reproductive Physiology of Fish. Austin, TX: University of Texas (1995). p. 129.

[56] Omoto N Maebayashi M Mitsuhashi E Yoshitomi K Adachi S Yamauchi K Effects of estradiol-17 and 17-methyltestosterone on gonadal sex differentiation in the F2 hybrid sturgeon, the bester. Fish Sci. (2002) 68:1047–54. 10.1046/j.1444-2906.2002.00531.x

[57] Setiawan AN Ozaki Y Shoae A Kazeto Y Lokman PM. Androgen-specific regulation of FSH signalling in the previtellogenic ovary and pituitary of the New Zealand shortfinned eel, *Anguilla australis*. Gen Comp Endocrinol. (2012) 176:132–43. 10.1016/j.ygcen.2011.12.041 22343137

[58] Hornung MW Jensen KM Korte JJ Kahl MD Durhan EJ Denny JS. Mechanistic basis for estrogenic effects in fathead minnow (*Pimephales promelas*) following exposure to the androgen 17α-methyltestosterone: conversion of 17α-methyltestosterone to 17α-methylestradiol. Aquat Toxicol. (2004) 66:15–23. 10.1016/j.aquatox.2003.06.004 14687976

[59] Piferrer F Zanuy S Carrillo M Solar II Devlin RH Donaldson EM Brief treatment with an aromatase inhibitor during sex differentiation causes chromosomally female salmon to develop as normal, functional males. J Exp Zool. (1994) 270:255–62. 10.1002/jez.1402700304

[60] Wennstrom KL Crews D. Making males from females: the effects of aromatase inhibitors on a parthenogenetic species of whiptail lizard. Gen Comp Endocrinol. (1995) 99:316–22. 10.1006/gcen.1995.1115 8536943

[61] Antonopoulou E Bornestaf C Swanson P Borg B. Feedback control of gonadotropins in Atlantic salmon, *Salmo salar*, male parr. I. Castration effects in rematuring and nonrematuring fish. Gen Comp Endocrinol. (1999) 114:132–41. 10.1006/gcen.1998.7249 10094866

[62] Akhavan SR Falahatkar B Ward JM Lokman PM 11-Ketotestosterone induces oocyte growth, but does not affect oocyte cytology in pre-vitellogenic captive beluga, Huso huso L. Comp Biochem Physiol B Biochem Mol Biol. (2019) 232:51–9. 10.1016/j.cbpb.2019.02.009 30831206

[63] Lokman PM George KA Divers SL Algie M Young G. 11-Ketotestosterone and IGF-I increase the size of previtellogenic oocytes from shortfinned eel, *Anguilla australis, in vitro*. Reproduction. (2007) 133:955–67. 10.1530/REP-06-0229 17616725

[64] Kohn YY Symonds JE Lokman PM. The effects of 11-ketotestosterone on ovarian physiology of previtellogenic captive hapuku (*Polyprion oxygeneios*). Comp Biochem Physiol A Mol Integr Physiol. (2013) 166:496–502. 10.1016/j.cbpa.2013.07.032 23948118

[65] Lubzens E Young G Bobe J Cerdà J Oogenesis in teleosts: how fish eggs are formed. Gen Comp Endocrinol. (2010) 165:367–89. 10.1016/j.ygcen.2009.05.022 19505465

[66] Nagler JJ Davis TL Modi N Vijayan MM Schultz I Intracellular, not membrane, estrogen receptors control vitellogenin synthesis in the rainbow trout. Gen Comp Endocrinol. (2010) 167:326–30. 10.1016/j.ygcen.2010.03.022 20346361

[67] Damsteegt EL Mizuta H Hiramatsu N Lokman PM. How do eggs get fat? Insights into ovarian fatty acid accumulation in the shortfinned eel, Anguilla australis. Gen Comp Endocrinol. (2015) 221:94–100. 10.1016/j.ygcen.2014.12.019 25660471

[68] Babin P Carnevali O Lubzens E Schneider W Molecular aspects of oocyte vitellogenesis in fish. In: Babin PJ, Cerdà JEL, editors. The Fish Oocyte: From Basic Studies to Biotechnological Application. Dordrecht: Springer (2007). p. 39–76.

[69] Divers SL McQuillan HJ Matsubara H Todo T Lokman PM. Effects of reproductive stage and 11-ketotestosterone on LPL mRNA levels in the ovary of the shortfinned eel. J Lipid Res. (2010) 51:3250–8. 10.1194/jlr.M009027 20713648 PMC2952565

[70] Mori T Matsumoto H Yokota H. Androgen-induced vitellogenin gene expression in primary cultures of rainbow trout hepatocytes. J Steroid Biochem Mol Biol. (1998) 67:133–41. 10.1016/S0960-0760(98)00099-5 9877213

[71] Kim BH Takemura A Kim SJ Lee YD. Vitellogenin synthesis via androgens in primary cultures of tilapia hepatocytes. Gen Comp Endocrinol. (2003) 132:248–55. 10.1016/S0016-6480(03)00091-1 12812772

[72] Fitzpatrick MS Gale WL Schreck CB. Binding characteristics of an androgen receptor in the ovaries of coho salmon, *Oncorhynchus kisutch*. Gen Comp Endocrinol. (1994) 95:399–408. 10.1006/gcen.1994.1139 7821777

[73] Ikeuchi T Todo T Kobayashi T Nagahama Y. cDNA cloning of a novel androgen receptor subtype. J Biol Chem. (1999) 274:25205–9. 10.1074/jbc.274.36.25205 10464240

[74] Sperry TS Thomas P Characterization of two nuclear androgen receptors in atlantic croaker: comparison of their biochemical properties and binding specificities 1. Endocrinology. (1999) 140:1602–11. 10.1210/endo.140.4.6631 10098494

[75] Sperry TS Thomas P. Identification of two nuclear androgen receptors in kelp bass (*Paralabrax clathratus*) and their binding affinities for xenobiotics: comparison with Atlantic croaker (*Micropogonias undulatus*) androgen receptors. Biol Reprod. (1999) 61:1152–61. 10.1095/biolreprod61.4.1152 10491657

[76] Tosaka R Todo T Kazeto Y Mark Lokman P Ijiri S Adachi S. Expression of androgen receptor mRNA in the ovary of Japanese eel, *Anguilla japonica*, during artificially induced ovarian development. Gen Comp Endocrinol. (2010) 168:424–30. 10.1016/j.ygcen.2010.05.005 20553719

[77] Leaños-Castañeda O Van Der Kraak G. Functional characterization of estrogen receptor subtypes, ERalpha and ERbeta, mediating vitellogenin production in the liver of rainbow trout. Toxicol Appl Pharmacol. (2007) 224:116–25. 10.1016/j.taap.2007.06.017 17662327

[78] Sabo-Attwood T Blum JL Kroll KJ Patel V Birkholz D Szabo NJ. Distinct expression and activity profiles of largemouth bass (*Micropterus salmoides*) estrogen receptors in response to estradiol and nonylphenol. J Mol Endocrinol. (2007) 39:223–37. 10.1677/JME-07-0038 17909263

[79] Katsu Y Kohno S Hyodo S Ijiri S Adachi S Hara A. Molecular cloning, characterization, and evolutionary analysis of estrogen receptors from phylogenetically ancient fish. Endocrinology. (2008) 149:6300–10. 10.1210/en.2008-0670 18635653 PMC2734497

[80] Latonnelle K Fostier A Le Menn F Bennetau-Pelissero C. Binding affinities of hepatic nuclear estrogen receptors for phytoestrogens in rainbow trout (*Oncorhynchus mykiss*) and siberian sturgeon (*Acipenser baeri*). Gen Comp Endocrinol. (2002) 129:69–79. 10.1016/S0016-6480(02)00512-9 12441116

[81] Nelson ER Habibi HR. Functional significance of nuclear estrogen receptor subtypes in the liver of goldfish. Endocrinology. (2010) 151:1668–76. 10.1210/en.2009-1447 20194729

[82] Pretorius E Arlt W Storbeck KH. A new dawn for androgens: novel lessons from 11-oxygenated C19 steroids. Mol Cell Endocrinol. (2017) 441:76–85. 10.1016/j.mce.2016.08.014 27519632

[83] Miller WL. Steroidogenesis: unanswered questions. Trends Endocrinol Metab. (2017) 28:771–93. 10.1016/j.tem.2017.09.002 29031608

[84] Bogart MH. Sex determination: a hypothesis based on steroid ratios. J Theor Biol. (1987) 128:349–57. 10.1016/S0022-5193(87)80077-2 3444342

